# Assessment of Health‐Related Quality of Life in Thai Endometrial Cancer Patients: A Comparative Analysis Using EQ5D Utility Scores Across States

**DOI:** 10.1002/cnr2.70139

**Published:** 2025-02-10

**Authors:** Vitcha Poonyakanok, Jitra Eagjeen, Tepnaree Kwangngoen, Pattara Leelahavarong, Natthakan Chitpim

**Affiliations:** ^1^ Department of Obstetrics and Gynaecology, Faculty of Medicine Siriraj Hospital Mahidol University Bangkok Thailand; ^2^ Siriraj Health Policy Unit, Faculty of Medicine Siriraj Hospital Mahidol University Bangkok Thailand

**Keywords:** endometrial cancer, EQ‐5D‐5L, quality of life

## Abstract

**Background:**

Rates of endometrial cancer, the sixth most common in women, are rising. HRQoL, reflecting health beyond clinical contexts, includes disabilities and daily functioning impacts. Measured by various tools such as EuroQoL‐5 Dimensions (EQ‐5D‐5L), it aids in economic evaluation of interventions.

**Aims:**

The purpose of this study was to analyze Health‐Related Quality of Life (HRQoL), measured by the EQ‐5D‐5L instrument, in different states of endometrial cancer (EC) patients.

**Methods and Results:**

We conducted a cross‐sectional study on EC patients who underwent follow‐up at Siriraj Hospital, Thailand, between January and June 2023. Patients were classified into five groups: early state, advanced state, curative state, locoregional recurrent state, and distant recurrent/progression state. Demographic and socioeconomic data were collected. EQ‐5D‐5L and visual analog scale instruments (EQ‐VAS) were used to compared between disease states. Descriptive statistics were used to summarize patient characteristics, and the Mann–Whitney U test (*p* ≤ 0.05) compared EQ‐5D‐5L scores across groups.

The study included 56 EC patients, with a mean age of 60.1 ± 10.9 years and a mean BMI of 26.8 ± 5.3 kg/m^2^. The EQ‐5D scores were as follows: 0.9055 (IQR 0.8193–0.9436) for the early state, 0.8308 (IQR 0.7997–0.8611) for the advanced state, 0.9235 (IQR 0.8521–0.9855) for the curative state, 0.9096 (IQR 0.6249–0.9577) for the locoregional recurrent state and 0.5778 (IQR 0.2884–0.8521) for the distant recurrent/progressive state. The median EQ‐VAS for each state was 70, 75, 82.5, 75, and 65, respectively. The EQ‐5D values had significantly deteriorated after distant metastasis/progression compared to curative states (*p*‐value = 0.003). Mobility and pain/discomfort appeared to be the two main concerns.

**Conclusion:**

The findings show the substantial negative impact of distant metastasis or disease progression on HRQoL. These findings will be used to guide future economic research in the field of endometrial cancer treatment.

## 1 Introduction

Endometrial cancer is the sixth most common cancer and the 13th most common cause of death among women worldwide [1]. From 2006 to 2012, the incidence rate increased by 10% or 2.5% each year [2]. In Thailand, endometrial cancer was the sixth cancer with a standardized age incidence rate of 7.6 cases per 100 000 Thai population and more than 4700 cases died in 2020 [1].

Endometrial cancer is diagnosed mainly in the early stages and in this stage has a very good prognosis with a 5‐year overall survival rate of 99.5% [3]. Due to improved diagnosis and treatment, patients would live longer. However, patients with endometrial cancer encounter a combination of treatment including surgery, chemotherapy, and radiation, with adverse events such as cystitis or proctitis from radiation or lymphedema from surgery, which decreases the quality of life. A previous HRQoL study of Thai endometrial cancer using QLQ‐C30 showed that adjuvant treatment with radiation or chemotherapy had negative impacts on HRQoL in endometrial cancer survivors [4]. It could imply that a late stage or a distant recurrence could result in a lower HRQoL.

HRQoL encompasses outcomes that extend beyond clinical contexts, reflecting the health aspects affected by the impact of diseases and treatments on disability and daily functioning. HRQoL could be measured by many instruments, including patient‐reported outcome measures. One of the most popular scales is the EuroQoL‐5 Dimensions (EQ‐5D‐5L), established in 1990 by the EuroQoL Group. It has been used in cancer patients, such as colorectal cancer [5].

Few studies [6, 7, 8, 9] have measured health utility in endometrial cancer, and most published research has focused on Western populations, including the United States, the United Kingdom, Germany, and one study from Australia. Furthermore, most of these studies [6, 8, 9] have used the EQ‐5D‐3L questionnaire, with only one study employing the EQ‐5D‐5L in this context [7]. These studies typically assessed patients in a single state of health, such as early‐stage or advanced‐stage endometrial cancer. To date, no studies have been conducted in developing countries, such as Thailand, and no research has assessed multiple stages of endometrial cancer using the EQ‐5D‐5L. Since the EQ‐5D‐5L is more detailed, sensitive, and reliable data compared to EQ‐5D‐3L, especially in detecting small but important changes in quality of life in the capture of HRQoL, this leaves a significant gap in our understanding of HRQoL and utility scores for endometrial cancer patients in Southeast Asia.

Furthermore, economic evaluation research is becoming increasingly important as it helps policy makers decide which interventions to implement and facilitates cost‐utility analysis. According to Thailand's Health Technology Assessment (HTA) guidelines, the EQ‐5D‐5L is recommended to determine utility scores [10]. These scores are a key component in calculating quality‐adjusted life years (QALYs), which are used to conduct cost‐effectiveness research. The objective of this research is to use the EQ‐5D‐5L tool to examine the utility scores of the health state of five different groups of stages of endometrial cancer in a tertiary hospital setting.

## 2 Methods

We conducted a cross‐sectional study at the Siriraj Hospital Faculty of Medicine, Mahidol University, Thailand, from January 2023 to June 2023. The eligible criteria included patients diagnosed with endometrial cancer (ICD 10 C54.1), histologically confirmed at Siriraj Hospital. Patients unable to communicate in Thai and those with secondary metastatic disease in the uterus were excluded. Staging was determined using the 2009 FIGO (The International Federation of Gynecology and Obstetrics) guidelines [11].

We categorized patients into five states: (1) early state (stage 1 and 2 endometrial cancer patients); (2) advanced state (stage 3 and 4 endometrial cancer patients); (3) curative state (patient who was evaluated for a clinically complete response through vaginal examination and/or radiographic examination); (4) locoregionally recurrent state: patient who had been curative and later experienced a recurrence in the pelvic area; and (5) distant recurrent/progression state: patient who had been curative and became recurrent outside the pelvic area or patient whose disease was progressing during treatment.

Patients gave their informed consent, after which a structured self‐administered questionnaire was used to collect demographic data, EQ‐5D‐5L, and EQ‐VAS information. Demographic and socioeconomic details included age, body mass index (BMI), parity, underlying diseases, residential area, marital status, level of education, occupation, health insurance, and family income. Cancer‐related information also included cell type, cancer stage, and cancer treatment.

For the administration of the questionnaire, patients who did not understand or needed help underwent a face‐to‐face interview. Our questionnaire utilized the EQ‐5D‐5L, developed by the EuroQoL Group in 2009 [12]. This questionnaire consisted of five dimensions: mobility, self‐care, usual activities, pain/discomfort and anxiety/depression, each with five levels indicating ‘slight moderate–severe’ problems, ‘no problems’, and ‘unable to do’. We used the Thai EQ‐5D‐5L value set to calculate the health utility scores. The value set converts the five‐dimension responses from the EQ‐5D‐5L into a single utility score on a scale from 0 to 1, with 0 representing death and 1 indicating full health. The EQ‐5D‐5L scores were recorded as a sequence of five numbers, such as ‘32123’. The optimal state was represented by 11 111, indicating ‘no problems’ across all domains, while the poorest state was denoted as 55 555, indicating ‘unable to do’ across all domains. The questionnaire was validated in its Thai version, with the second‐best value recorded as 0.9436 for the 11 121 health state, and the worst state (55555) value being −0.4212 [13].

We also asked patients for EQ‐VAS. The EQ‐VAS is a standard visual analog scale to assess your current overall health status. It is a scale ranging from 0 to 100, where 0 indicates the worst state and 100 indicates the best state. The patient has to mark the scale on his own visual scale of his current health status [14].

### 2.1 Statistical Analysis

All statistical analyzes were performed using the Statistical Package for the Social Science software for window (SPSS) version 18. The EQ‐5D‐5L score was transformed into a utility score by conversion from its five‐number health states employing an additive formula that incorporates coefficients and a constant determined through the Thai value set, developed based on a recent national household survey conducted within the Thai general population [13]. Continuous data had already tested normality by the Kolmogorov–Smirnov test. Demographic and socioeconomic data were presented as numbers (percentage), mean ± SD, and median (IQR). The EQ‐5D and EQ‐VAS score was presented as a median (IQR). Comparisons of the EQ‐5D and EQ‐VAS scores between health states and curative state were analyzed using the Mann–Whitney *U* test with a *p*‐value ≤ 0.05 indicating statistical significance between groups.

## 3 Results

In this study, a total of 56 patients were enrolled and a self‐questionnaire was completed. The mean age was 60 years (range 34–82 years), and the mean BMI was 27 kg/m^2^. The cohorts of patients were stratified into five distinct groups based on their clinical status: 22 patients in an early stage, six in an advanced stage, 16 in a curative stage, and six each in the locoregionally recurrent and distant recurrent/progressive stages. The endometrioid subtype was predominant in 77% of the cases, while the high‐grade serous subtype constituted 11%. Approximately three‐quarters of the patients received adjuvant therapy; most of them were treated by radiotherapy alone or chemotherapy alone. More demographic details are provided in Table 1.

**TABLE 1:** Demographic data for each state of 56 endometrial cancer patients.

	All (*n* = 56)	Early (*n* = 22)	Advanced (*n* = 6)	Curative (*n* = 16)	Locoregional recurrence (*n* = 6)	Distant recurrence/progression (*n* = 6)
Age (years) (mean ± SD)	60.1 ± 10.9	58.9 ± 11.2	68.7 ± 6.9	59.1 ± 10.3	61.5 ± 8.9	62.8 ± 15.3
BMI (kg/m^2^) (mean ± SD)	26.8 ± 5.3	25.8 ± 3.8	25.2 ± 4.1	27.4 ± 6.0	26.8 ± 6.5	30.6 ± 7.9
Parity (*n*, %)						
Nulliparous	19 (33.9)	8 (36.4)	2 (33.3)	6 (37.5)	2 (33.3)	1 (16.7)
Multiparous	37 (66.1)	14 (63.6)	4 (66.7)	10 (62.5)	4 (66.7)	5 (83.3)
Cell type (*n*, %)						
Endometrioid Gr.1	22 (39.3)	9 (40.9)	2 (33.3)	7 (43.8)	3 (50)	1 (16.7)
Endometrioid Gr.2	16 (28.6)	8 (36.4)	1 (16.7)	6 (37.5)	1 (16.7)	
Endometrioid Gr.3	5 (8.9)	1 (4.5)	1 (16.7)	0 (0)	1 (16.7)	2 (33.3)
High grade serous	6 (10.7)	1 (4.5)	1 (16.7)	1 (6.3)	1 (16.7)	2 (33.3)
High grade	1 (1.8)	1 (4.5)	0 (0)	0 (0)	0 (0)	0 (0)
Clear cell	2 (3.6)	0 (0)	0 (0)	2 (12.5)	0 (0)	0 (0)
Carcinosarcoma	3 (5.4)	1 (4.5)	1 (16.7)	0 (0)	0 (0)	1 (16.7)
Mixed	1 (1.8)	1 (4.5)	0 (0)	0 (0)	0 (0)	0 (0)
Stage (*n*, %)						
1	33 (58.9)	20 (90.9)	0 (0)	9 (56.3)	4 (66.7)	0 (0)
2	3 (5.4)	2 (9.1)	0 (0)	1 (6.3)	0 (0)	0 (0)
3	18 (32.1)	0 (0)	6 (100)	6 (37.5)	2 (33.3)	4 (66.7)
4	2 (3.6)	0 (0)	0 (0)	0 (0)	0 (0)	2 (33.3)
Adjuvant treatment (*n*, %)						
None	15 (26.8)	9 (40.9)	0 (0)	6 (37.5)	0 (0)	0 (0)
Chemotherapy	16 (28.6)	3 (13.6)	4 (66.7)	5 (31.3)	0 (0)	4 (66.7)
Radiotherapy	17 (30.4)	9 (40.9)	0 (0)	2 (12.5)	5 (83.3)	1 (16.7)
Combined	8 (14.3)	1 (4.5)	2 (33.3)	3 (18.8)	1 (16.7)	1 (16.7)
Underlying disease (*n*, %)						
Diabetes mellitus	13 (23.2)	4 (18.2)	3 (50)	3 (18.8)	0 (0)	3 (50)
Hypertension	25 (44.6)	7 (31.8)	4 (66.7)	8 (50)	3 (50)	3 (50)
Dyslipidemia	19 (33.9)	9 (40.9)	3 (50)	5 (31.3)	1 (16.7)	1 (16.7)

Abbreviations: BMI, body mass index; Gr, Group; SD, standard deviation.

The socioeconomic characteristics of the study participants, including marital status, level of education, occupation, and health insurance status, are summarized in Table 2. Approximately 50% of the patients reported being married, while 27% identified as single. Half of the participants were retired. Among the study population, 21 individuals (38%) had a bachelor's degree. The median annual household income was 12 000 USD (IQR, 6171–20 571 USD).

**TABLE 2:** Socioeconomic data for each state of 56 endometrial cancer patients.

	All (*n* = 56)	Early (*n* = 22)	Advance (*n* = 6)	Curative (*n* = 16)	Locoregional recurrence (*n* = 6)	Distant recurrence/progression (*n* = 6)
Residential area (*n*, %)						
Urban	26 (46.4)	10 (45.5)	5 (83.3)	7 (43.8)	1 (16.7)	3 (50)
Rural	30 (53.6)	12 (54.5)	1 (16.7)	9 (56.2)	5 (83.3)	3 (50)
Marital status (*n*, %)						
Single	15 (26.8)	6 (27.3)	1 (16.7)	5 (31.3)	2 (33.3)	1 (16.7)
Married/cohabitating	28 (50)	12 (54.5)	3 (50)	5 (31.3)	3 (50)	5 (83.3)
Widow	11 (19.6)	3 (13.6)	2 (33.3)	5 (31.3)	1 (16.7)	0 (0)
Divorced/separated	2 (3.6)	1 (4.5)	0 (0)	1 (6.3)	0 (0)	0 (0)
Education (*n*, %)						
1. Never attended school	1 (1.8)	0 (0)	1 (16.7)	0 (0)	0 (0)	0 (0)
2. Primary school	20 (35.7)	9 (40.9)	3 (50)	3 (18.8)	3 (50)	2 (33.3)
3. High school/vocational certificate	9 (16.1)	5 (22.7)	0 (0)	4 (25)	0 (0)	0 (0)
4. Diploma/high vocational certificate	5 (8.9)	0 (0)	1 (16.7)	2 (12.5)	1 (16.7)	1 (16.7)
5. Bachelor or equivalent/postgraduate degree	21 (37.5)	8 (36.4)	1 (16.7)	7 (43.8)	2 (33.3)	3 (50)
Occupation (*n*, %)						
1. Agriculture/fisheries	3 (5.4)	1 (4.5)	1 (16.7)	0 (0)	1 (16.7)	0 (0)
2. Business owner	11 (19.6)	5 (22.7)	1 (16.7)	3 (18.8)	1 (16.7)	1 (16.7)
3. Laborer	5 (8.9)	4 (18.2)	0 (0)	1 (6.3)	0 (0)	0 (0)
4. Government/state enterprise officer	7 (12.5)	3 (13.6)	0 (0)	2 (12.5)	1 (16.7)	1 (16.7)
5. Retired/unemployed	28 (50)	8 (36.4)	4 (66.7)	9 (56.3)	3 (50)	4 (66.7)
6. Other	2 (3.6)	1 (4.5)	0 (0)	1 (6.3)	0 (0)	0 (0)
Health insurance (*n*, %)						
1. Universal Coverage Scheme	31 (55.4)	10 (45.5)	5 (83.3)	10 (62.5)	4 (66.7)	2 (333)
2. Social Security Scheme	4 (7.1)	3 (13.6)	0 (0)	0 (0)	1 (16.7)	0 (0)
3. Civil Servant Medical Benefit Scheme	19 (33.9)	7 (31.8)	1 (16.7)	6 (37.5)	1 (16.7)	4 (66.7)
4. Out of pocket	2 (3.6)	2 (9.1)	0 (0)	0 (0)	0 (0)	0 (0)
Household income per year (USD)^a^ (median, IQR)	12000.0 (6171.4–20571.4)	13200.0 (6857.1c26914.3)	9600.0 (8160.0–12000.0)	10285.7 (3480.0–13628.6)	11417.1 (5100.0–24000.0)	31714.3 (3582.9–58628.6)

^a^ 35 Thai Baht = 1 US$, 1 missing data.

**FIGURE 1:**
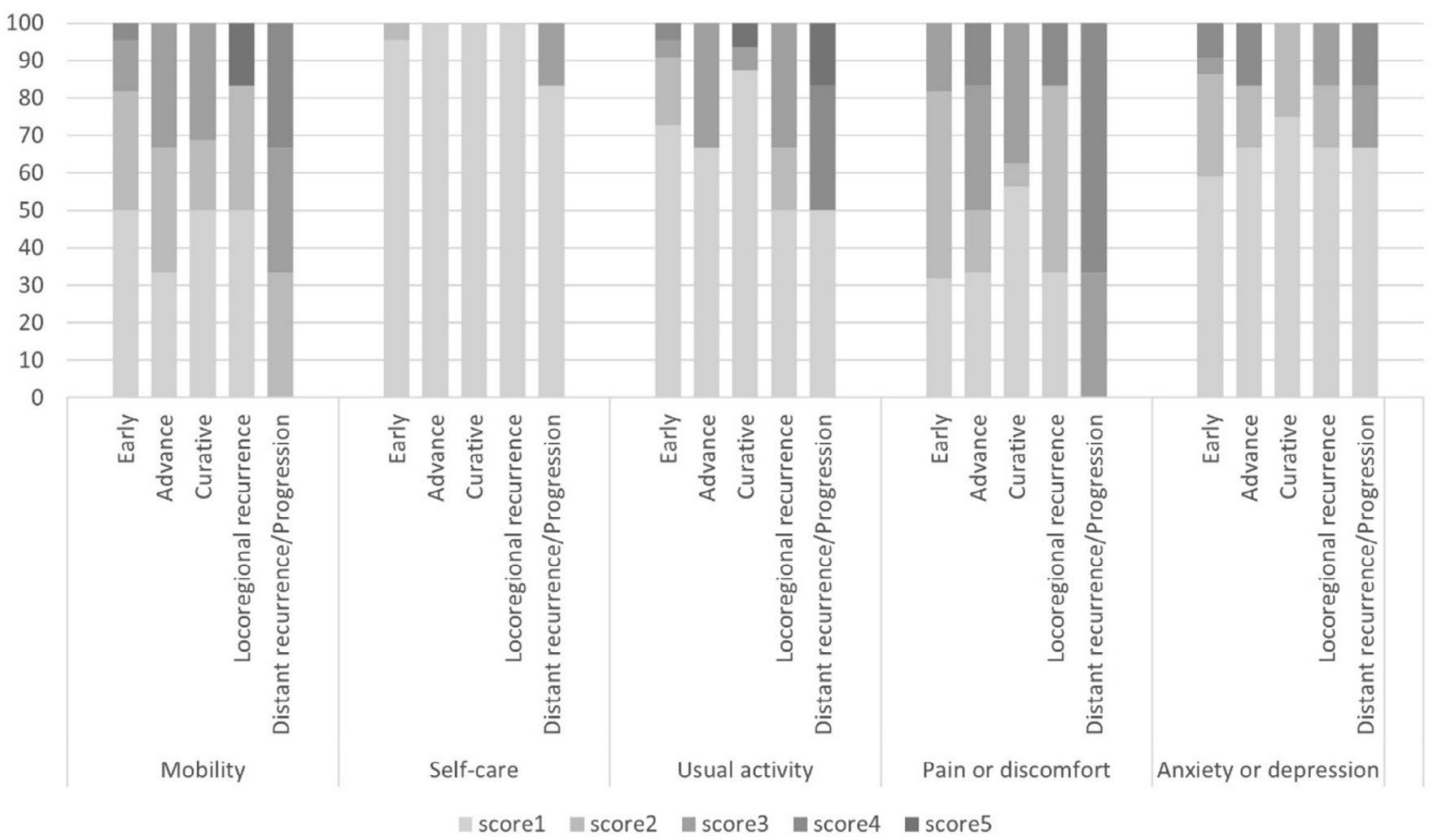
Column chart of the EQ‐5D‐5L score in five domains between each state of the patients.

Examination of the EQ‐5D‐5L data revealed significant challenges in the domains of mobility and pain/discomfort among endometrial cancer patients. In particular, individuals in the distant recurrent/progression (metastasis) state exhibited an obvious trend of encountering more difficulties compared to other states. In contrast, patients in the curative state demonstrated a tendency to experience fewer problems in these domains. Figure 1 visually represents these findings through a column graph depicting the percentage of utility score range from 1 to 5 in each domain between each state of the patients.

The median EQ‐5D‐5L score of patients in the early state, advanced state, curative state, locoregionally recurrent state and distant recurrent/progression state was 0.90 (IQR, 0.82–0.94), 0.83 (IQR, 0.80–0.86), 0.92 (IQR, 0.85–0.99), 0.91 (IQR, 0.62–0.96), and 0.58 (IQR, 0.29–0.85), respectively. In the early state, three patients (13.6%) reported a full health EQ‐5D‐5L score of 1.0, as did one patient (16.7%) in the advanced state, four patients (25%) in the curative state, and one patient (16.7%) in the locoregionally recurrent state. Most patients demonstrated relatively good EQ‐VAS scores, with median scores ranging between 75 and 83, except for those in the distant recurrent/progressive state, who had a median score of 65 (IQR, 43–75).

Patients in the distant recurrent/progression state exhibited statistically significant lower utility values in both EQ‐5D‐5L (*p* = 0.003) and EQ‐VAS (*p* = 0.015) compared to those in the curative state. However, no significant differences were observed between the other states. Detailed comparative data between the various states can be found in Table 3.

**TABLE 3:** Comparative data on EQ‐5D‐5L and EQ‐VAS scores between disease states.

	Median EQ‐5D (IQR)	*p*‐value	Median EQ‐VAS (IQR)	*p*‐value
Curative	0.9235 (0.8521–0.9855)	Ref	83 (80–95)	Ref
Early	0.9055 (0.8193–0.9436)	0.667	80 (69–90)	0.152
Advance	0.8308 (0.7997–0.8611)	0.128	75 (65–93)	0.311
Locoregional recurrence	0.9096 (0.6249–0.9577)	0.824	75 (65–97)	0.407
Distant recurrence/Progression	0.5778 (0.2884–0.8521)	**0.003**	65 (43–75)	**0.015**

*Note*: The bold formatting was used to highlight only the significant differences to ensure clarity and readability in the table.

Abbreviations: ED‐VAS, EuroQoL visual analog scale; IQR, interquartile range; Ref, reference.

## 4 Discussion

This study explored HRQoL among Thai endometrial cancer patients in five different stages consisting of early stage, advanced stage, cure stage, locoregional recurrence stage, and distant recurrence or progression stage. Analysis of the EQ‐5D‐5L data revealed substantial challenges in the mobility and pain/discomfort domains among endometrial cancer patients. In particular, individuals in the distant recurrent/progressive state faced more pronounced difficulties, indicating the profound impact of disease progression on HRQoL. In contrast, those in the curative state exhibited a trend towards fewer issues in these domains, indicative of the potential improvement in HRQoL after treatment.

When comparing the EQ‐5D questionnaire with other cancer‐specific instruments such as the Functional Assessment of Cancer Therapy–General (FACT‐G) and the Treatment of Cancer Quality of Life questionnaire (QLQ‐C30) in various cancers (i.e., lung, breast and colorectal), the convergent validity showed that FACT‐G had a strong correlation with the dimensions of EQ‐5D [15], particularly in areas that were theoretically similar to the scales of the QLQ‐C30 [16]. Furthermore, EQ‐5D‐5L outperformed EQ‐5D‐3L, demonstrating better discriminatory power, higher test–retest reliability, reduced ceiling effects, stronger convergent validity and higher relative efficiency. Notably, the strength of correlation for EQ‐5D‐5L was higher than for EQ‐5D‐3L in all dimensions and hypotheses, indicating that the EQ‐5D‐5L more effectively captures similar concepts of health‐related quality of life and provides stronger correlations with other established instruments [15, 16, 17].

The disparity in the EQ‐5D‐5L scores between different stages of cancer corroborates these findings. Patients in the curative state consistently demonstrated higher median EQ‐5D‐5L scores compared to those in other stages, while patients in the distant recurrent/progressive state exhibited significantly lower scores. This emphasizes the influential role of disease status on HRQoL outcomes. Furthermore, the EQ‐VAS scores showed a similar trend, with patients in the distant recurrent/progressive state reporting significantly lower scores compared to those in the curative state. This disparity in the EQ‐VAS scores highlights the subjective perception of overall health status and its correlation with disease progression, mirroring the findings of EQ‐5D‐5L.

The incidence of endometrial cancer is increasing and the choice of treatment depends on the stage of the disease, ranging from surgical intervention alone to a combination of surgery with radiotherapy or chemotherapy, or chemotherapy as a standalone option. Each treatment modality has a distinct impact on the quality of life of patients. According to Joly et al. [18], surgery, particularly within the initial 6 months after diagnosis, significantly affects the physical and functional domains of quality of life.

Early stage endometrial cancer is associated with a favorable prognosis, and patients tend to experience extended survival with the implementation of appropriate surgical treatment and adjuvant radiation. However, the study by Zhu et al. [19] suggests a potential downside to the use of radiation therapy in early‐stage endometrial cancer. According to their findings, radiation therapy can significantly worsen quality of life (QOL) in patients undergoing staged surgery. This underscores the importance of carefully considering the potential impact of treatment modalities on the quality of life of patients.

In advanced stage endometrial cancer, patients often receive adjuvant chemotherapy, a treatment approach associated with potential long‐term side effects such as polyneuropathy or cardiac dysfunction. These findings are consistent with the results of a cross‐sectional study conducted by Sivapornpan et al. [4]. Their study, which compared surgery alone versus surgery with adjuvant treatment in endometrial cancer survivors, reported that adjuvant treatment with radiation or chemotherapy had negative impacts on the quality of life of individuals who had undergone endometrial cancer treatment. Our study aligns with these considerations, revealing that patients in the early stages exhibit a notably high EQ‐5D score of 0.91. In contrast, those in advanced stages experience a substantial impact on the quality of life (QOL), with an EQ‐5D score of 0.83, particularly in the domain of mobility.

For patients experiencing recurrent disease, those with only locoregional recurrence demonstrated a notably high QOL. On the contrary, patients with distant recurrence experienced a significant decline in quality of life in all domains, particularly mobility and pain/discomfort. The substantial drop in quality of life, especially in domains related to mobility and pain/discomfort, highlights the challenges faced by people dealing with distant recurrence, emphasizing the need for targeted interventions and comprehensive support for this specific population of patients.

Several studies have investigated various aspects of the QOL in endometrial cancer patients, shedding light on different factors that influence their well‐being. In a study by Dobrzycka et al. (2017) [20], they reported that endometrial cancer survivors exhibited a QOL comparable to a healthy cohort 3 years after surgery. However, the introduction of adjuvant radiotherapy was found to influence QOL, indicating the subtle impact of treatment modalities on survivors' well‐being. A longitudinal study using SF‐36 after endometrial cancer surgery, conducted by Chen et al. [21], revealed that quality of life in the second and third months after the operation was notably better than in the first month. Furthermore, higher family income emerged as a factor associated with a better quality of life after surgery, emphasizing the socioeconomic dimension in postoperative well‐being. Ferguson et al. [22] reported in their study that minimally invasive approaches resulted in an improved quality of life beyond the short‐term postoperative period, with benefits observed up to 12 weeks after surgery [14]. This contrasts with the findings of Salehi et al. [23], who observed that laparotomy and robot‐assisted surgery conferred similar health‐related quality of life 12 months after complete staging for high‐risk endometrial cancer [15].

Interventions aimed at improving QOL in endometrial cancer patients have shown promise, as demonstrated in the study by Robertson et al. [24] In their research, a telephone‐based physical activity intervention was implemented and the findings revealed a positive association between increased physical activity and improvements in various aspects of QOL; general health, role limitation due to physical health, pain, and somatic distress.

Taking into account the outcome of the clinical study, the HRQoL study showed the humanistic impact of disease, as well as economic considerations, the study by Joly et al. [18] advocated the inclusion of quality of life (QOL) evaluations in every clinical trial for endometrial cancer. We believe that such assessments, which encompass pain, fatigue, emotional well‐being, and social functioning, are essential for a comprehensive understanding of the patient's experience. Moreover, we emphasize the need to evaluate financial toxicity, recognizing the potential economic burdens placed on patients and their families during cancer treatment. For future studies, exploring the HRQoL of endometrial cancer at each stage could contribute significantly to the economic evaluation of preventive measures and treatment strategies. This evidence could inform policy makers about cost‐effective interventions that could be included in the country's health benefit packages.

The study has certain limitations. First, self‐administration of the questionnaire by patients introduces the possibility of errors due to subjective factors. Second, the small sample size prevented the performance of a regression analysis of clinical characteristics. Finally, it is important to note that the data was derived from a single tertiary hospital. Extrapolating the results to a broader population should be approached with caution.

## Author Contributions

V.P., N.C.: conceptualization. V.P., N.C.: methodology. T.K, J.E.: data curation. V.P., P.L.: formal analysis. V.P.: writing – original draft. V.P., N.C., P.L.: writing – review and editing. V.P.: visualization. N.C.: supervision. P.L.: funding acquisition. All authors had full access to the data in the study and take responsibility for the integrity of the data and the accuracy of the data analysis.

## Ethics Statement

This study received approval from the Mahidol University Multi‐Faculty Cooperative IRB No. 205/2022 (Protocol No. 1108/2564 IRB3). The ethic compliances with international Guidelines for Human Research Protection such as Declaration of Helsinki, The Belmont Report, CIOMS Guidelines and International Conferences on Harmonization in Good Clinical Practice (ICH‐GCP).

## Consent

Informed consent has been obtained from the involved patients.

## Conflicts of Interest

The authors declare no conflicts of interest.

## Data Availability

Data supporting the findings of this study are available on request from the corresponding author. Data are not publicly available due to privacy or ethical restrictions.
